# On the relationship between the social brain, social connectedness, and wellbeing

**DOI:** 10.3389/fpsyt.2023.1112438

**Published:** 2023-02-24

**Authors:** M. Justin Kim, Sunhae Sul

**Affiliations:** ^1^Department of Psychology, Sungkyunkwan University, Seoul, Republic of Korea; ^2^Center for Neuroscience Imaging Research, Institute for Basic Science, Suwon, Republic of Korea; ^3^Department of Psychology, Pusan National University, Busan, Republic of Korea

**Keywords:** happiness, wellbeing, social connectedness, social brain, fMRI, social neuroscience

## Abstract

The emergence of social neuroscience in the past two decades has offered a useful neurocognitive framework for understanding human social behavior. Of importance, social neuroscience research aimed to provide mechanistic explanations for the established link between wellbeing and social behavioral phenomena–particularly those reflective of social connectedness. Here, we provide an overview of the relevant literature focusing on recent work using functional magnetic resonance imaging (fMRI). In general, fMRI research demonstrated that aspects of social connectedness that are known to either positively (e.g., social acceptance) or negatively (e.g., social isolation) impact wellbeing also modulated the activity of subcortical reward system accordingly. Similar modulatory influence was found for the activity of other brain regions such as the medial prefrontal cortex, which are typically regarded as components of the “social brain” that support a wide range of functions related to social cognition and behavior. Elucidating such individual differences in brain activity may shed light onto the neural underpinnings of the link between social connectedness and wellbeing.

## Introduction

Social neuroscience is an interdisciplinary field of research that uses cognitive neuroscience approaches to answer questions pertaining to social psychology (1). Of the wide range of research topics that fall under the broad category of social neuroscience, one major line of work focuses on elucidating the effects of social connectedness and their neural underpinnings. These include how we successfully (e.g., social acceptance) or fail to connect to others (e.g., social exclusion), feeling disconnected from other people (e.g., social isolation), and being benevolent to others (e.g., prosocial behavior) that may in turn promote social connectedness. This is of particular importance, as social connectedness has been recognized to have direct physical and mental health implications (2). In the past two decades, in conjunction with the technological advances of functional magnetic resonance imaging (fMRI) methods, social neuroscience research on these topics has made significant progress. Here, we highlight a series of such fMRI studies that attempt to provide a mechanistic explanation between social connectedness with wellbeing and happiness. To achieve this, we provide a summarized account of each of the major factors that contribute to either promoting or preventing social connectedness, which include (1) social isolation and loneliness; (2) social exclusion and rejection; (3) social bonding and acceptance; and (4) prosocial behavior.

## Social connectedness, social brain, and wellbeing

### Social isolation and loneliness

Feeling disconnected from other people can be detrimental to psychological and physical wellbeing (3–5). Perceived social isolation, or loneliness (6), has been suggested to increase the risk for premature mortality (7) and suicide (8). Given the widespread negative impact of loneliness on wellbeing, social neuroscientists were motivated to investigate its effects on the brain, mostly focused on the functional responsivity of the social brain areas. Early neuroimaging work tested the extent to which loneliness modulated regional brain activity in response to socioemotional stimuli. Motivated by findings from social neuroscience research highlighting ventral striatum (VS) responsivity to socially rewarding situations or states such as cooperation (9), social comparison (10), or romantic love (11), one such study leveraged individual differences in loneliness and probed brain activity using pleasant and unpleasant social pictures (12). This study found that lonely individuals show less activity of the VS to pleasantly depicted social stimuli, which provided neural evidence that they were less rewarded by social stimuli (12).

This idea that the activity of the subcortical reward systems being dampened toward social stimuli as a function of loneliness has received some support from subsequent fMRI studies (13, 14). For example, in lonely individuals, activity of the VS was decreased when seeing faces of strangers (13). Interestingly, their VS activity increased when seeing faces of close others, which was interpreted as reflecting a possible desire for social reconnection. Another study has corroborated and expanded the initial findings by Cacioppo et al. (12), such that lonely individuals showed blunted activity of the VS during interpersonal trust decisions in a trust game (14). Collectively, fMRI investigation of loneliness suggests that the VS–a core component of the subcortical reward system–is affected by perceived social isolation, which in turn may negatively influence psychological wellbeing [but see also (15) for a failed replication of these effects in younger and older adults]. It is noteworthy that blunted reward-related VS activity is often associated with major depressive disorder (16), and predicts the emergence of depressive symptoms across development (17). Given the link between loneliness and depression (4), mechanistically, the negative mental health consequences of social isolation may be rooted in a dysfunctional VS, especially toward socially rewarding stimuli.

More recent neuroimaging work on loneliness has shifted its focus from the VS to other brain regions and systems related to socioemotional processing including the medial prefrontal cortex (MPFC) and amygdala (18), and other social brain regions such as the temporoparietal junction (TPJ) and precuneus that support higher-order social cognitive abilities (19). One study found that lonely individuals exhibited diminished functional connectivity between the anterior insula and precuneus, which corresponded to decreased affective responsiveness to positive social interactions (14). In line with the prediction that the social brain areas are impacted by loneliness, the anterior insula and precuneus are both centrally involved in social cognitive processing, such as trustworthiness decisions from faces (20) and self-referential operations (21), respectively.

Other fMRI studies have targeted the MPFC as a potential site that is negatively affected by loneliness, either as an isolated brain region or a part of a larger functional network (14, 22). These studies leveraged the MPFC for its well-known functional significance in self/other computations (23–25), which in turn may contribute to the representation of social connectedness and naturally, social isolation. This indeed appears to be the case, as the MPFC may serve to chart a map of one's friendships (26) and to keep tabs on the social network positions of others (27). Using a self- and other-reflection task during an fMRI scan, Courtney and Meyer (22) observed that the MPFC keeps separate neural representations for the self, social network members, and familiar individuals who exist outside of one's social network (i.e., celebrities). Of relevance, lonely individuals were characterized by an altered pattern of self-other mapping in the MPFC, such that loneliness attenuated the similarity between the neural representation of the self vs. others (22). In other words, the neural representation between the self and others in the MPFC were notably dissimilar in lonely individuals, perhaps reflecting their socially isolated psychological state.

Recent fMRI work has begun to examine the neural underpinnings of loneliness using macroscale functional networks (28, 29). While these usually take on the form of a data-driven approach, the default mode network, which typically includes many of the aforementioned cortical midline structures such as the MPFC and precuneus, has been the focal point of such investigations for its known role in self-referential processing (30). In an analysis of a very large study sample exceeding 38,000 unique resting state fMRI data, lonely individuals showed greater functional connectivity within the default mode network compared to other canonical functional networks (29). Interestingly, these findings are interpreted such that lonely individuals, driven by the absence of social experiences, may be more inclined to recruit the default mode network to enhance the mental stimulation of inner social events *via* mentalizing and imagination (29). Adopting a connectome-based predictive framework (31), another study identified a functional network model for loneliness that consisted of the MPFC and amygdala, among other brain regions (28). Considering the recent findings that elucidate the manner in which whole brain functional connectomes are associated with individual differences in wellbeing (32), loneliness may impact brain function on a large-scale network level, beyond isolated brain regions.

### Social exclusion and rejection

Social exclusion is the experience of being rejected by others, which compromises wellbeing in multiple ways (33, 34). Being socially rejected is often described to be hurtful, and this social pain has been a focal point of research on wellbeing, as it is suggested to be more easily reexperienced and longer lasting than physical pain (35), and have detrimental effects on mental and physical health (36). In social neuroscience research, the feeling of social exclusion is often experimentally induced to the participants by having them engage in a Cyberball task. Cyberball is a computer game that involves tossing and receiving a digital ball to and from other virtual players (37). Cyberball is deliberately designed to first establish a feeling of social inclusion by having the ball tossed around among all players, including the participant. Then, the other players stop throwing the ball to the participant and passes the ball only amongst themselves, generating a situation in which the participant feels socially excluded. The first study to use Cyberball in conjunction with fMRI found increased activity of the dorsal anterior cingulate cortex (dACC) and anterior insula in response to social exclusion (38). Anterior insula activity was linked to negative emotions experienced during Cyberball, both of which were modulated by emotional support (39). The dACC findings aligned with previous work on the neural representations of physical pain (40, 41), and thus these results were suggested to provide neural evidence that social exclusion and rejection signal pain. Following the initial report by Eisenberger et al. (38), dACC activity has been observed in a number of fMRI studies employing Cyberball (42, 43) and other experimental paradigms designed to emulate social exclusion scenarios (44, 45).

However, a recent quantitative meta-analysis of Cyberball fMRI studies suggests otherwise (34). Surprisingly, a voxel-based meta-analysis of 53 Cyberball fMRI studies that included 1,817 participants revealed that, in contrast to the suggestions from earlier work, the dACC did not show reliable activity to social exclusion–in fact, this meta-analysis found that only fewer than 15% of the studies reported dACC activity (34). Instead, this meta-analysis demonstrated that the most reliable activity to social exclusion was found in the ventral ACC (vACC) and posterior cingulate cortex (PCC), both of which are key components of the default mode network (19). When the resulting meta-analysis map was decoded using Neurosynth (46), the most relevant functions associated with social exclusion-induced brain activity patterns included self-referential processes, mentalizing and emotional valence (34). As it stands, the experience of social exclusion, at least for those generated by Cyberball, appears to primarily engage brain activity on a large-scale functional network level (i.e., default mode network), rather than the dACC in isolation.

### Social bonding and acceptance

If the need to belong is indeed a fundamental human motivation, it follows then our brain finds social bonding and acceptance to be positive or appetitive, thereby reinforcing behaviors that are more likely to lead to such outcomes (47). The opposite–that is, the possibility that our brain is designed to processes social exclusions and rejections as negative or aversive events and its health implications–has been discussed elsewhere in this review. Here, we focus on the neural underpinnings of social acceptance–that is, other people's desire to include you in their social groups (48).

Supporting the prediction that the experience of social acceptance is fundamentally positive/appetitive, fMRI studies have found that reward system was consistently activated when being accepted or obtaining other socially desirable outcomes. Specific brain regions included the VMPFC, vACC and VS (49, 50). Such interpretations are based on the idea that whatever engages these brain regions are rewarding, or at least treated as rewarding stimuli at the neural level. Indeed, it is well-known that rewards such as food and money activate the reward systems in the brain (51, 52). Of relevance, fMRI research found converging evidence for an overlapping representation of non-social and social reward in the VS and VMPFC (53), with the latter also being suggested as a key brain structure that is commonly engaged by personal and vicarious reward (54). In other words, the VS and VMPFC, and the reward system in general, are responsive to socially rewarding stimuli including social acceptance. For example, being liked (55) and receiving good reputation (50), both of which could be considered as proxies for increased likelihood of social acceptance, activate the reward system.

In scenarios that involve explicit interpersonal acceptance vs. rejection, the vACC is suggested to be responsive to being accepted–specifically, the positive valence of the social outcome (56). In the context of romantic acceptance vs. rejection, in addition to dACC and anterior insula being responsive to both acceptance and rejection, the striatum was more active when being accepted (57). This pattern of results was not specific to romantic acceptance, as these findings were strikingly similar to those of another fMRI study in which the participant's profile received social feedback. When the profile was liked (i.e., socially accepted), a corresponding increase in striatal activity was observed (58). Taken together, neuroimaging research consistently suggests that social acceptance is processed in the brain as if it is inherently rewarding and desirable. It is noteworthy that this is in contrast to the social exclusion literature, where the relationships between social exclusion, neural representation of pain, and dACC function are disagreed upon (59, 60).

### Prosocial behavior

Prosocial behavior can influence one's health and wellbeing by promoting positive social relationships and social resources (61–63). Prosocial individuals are more likely to receive social support (64) than less prosocial individuals and the concomitant other-regarding emotion such as compassion facilitates social connectedness (54, 65). Direct emotional benefits of prosocial behavior have also been reported. For example, participants who engaged in voluntary prosocial spending experienced greater increase in subjective wellbeing than those who spent money for themselves [for a review and replication, see Aknin et al. (66)]. Even a small act of kindness such as writing a note of appreciation decreased loneliness and improved mood (67). Moreover, prosocial behavior improves eudaimonic wellbeing [i.e., well-lived life or meaning of life; (68, 69)] and immune system (70, 71). A recent genomic study showed that volunteering had positive impact on immune cell gene regulation and the magnitude of this effect was positively correlated with the magnitude of increase in eudaimonic happiness after volunteering (72).

In an fMRI study examined the association between generosity and happiness (73), participants who spent money for others for 4 weeks, compared to those who spent money for themselves, became more generous and happier and showed greater TPJ-VS connectivity during a prosocial decision-making task. Although there is a paucity of work directly testing the neural underpinnings that link prosocial behavior and wellbeing, a considerable number of studies imply the hedonic benefits of prosociality. Earlier fMRI studies showed that making charitable donations (74, 75) and experiencing reciprocal cooperation (9, 76) were associated with the neural activity of the reward system comprising the VS, medial orbitofrontal cortex, subgenual ACC (sgACC), and VMPFC, indicating that prosocial outcomes have similar hedonic qualities to the reward given to self. The overlap between the neural mechanisms of self-regarding and other-regarding decisions has been consistently reported in ensuing studies (24, 77, 78). For instance, Sul and colleagues (24) used a prosocial learning task in which participants could gain reward for themselves of another person to examine the rewarding quality of helping others. The neural activity of the VMPFC and sgACC was correlated with the subjective value for both self-regarding and other-regarding decisions. However, the overlap between the value computation for self- and other-regarding learning was modulated by individual differences in prosociality. That is, the self-other overlap was found only among prosocial participants who placed greater value on the other-regarding choices, whereas proself participants showed clear distinction between self and other. Moreover, neural activity in the inferior frontal gyus (IFG) was increased when proself individuals made decisions for other than for self, and the magnitude of increase in the IFG activity was positively correlated with the reduced frontostriatal functional connectivity during the other-regarding compared to the self-regarding learning. Similar findings were observed in a later fMRI study on prosocial learning (78), which also reported the involvement of the VMPFC in the other-regarding valuation and the VMPFC-TPJ connectivity in the choices for others. In addition, corroborating the fMRI evidence for the involvement of cognitive control system, gray matter volume of DLPFC and prosocial decisions were negatively correlated among prosocial individuals, while the opposite pattern was found for proself individuals (79). Such modulatory effects of individual differences in prosocial propensity [e.g., trait empathy, (80)] and culture (81) were found in other fMRI studies as well.

A recent meta-analysis compared fMRI studies on altruistic and strategic prosocial decisions and revealed common and distinct neural correlates for the two types of decisions (82). This study sorted 36 fMRI studies involving various giving behaviors into altruistic prosocial decision (i.e., generous decision without extrinsic rewards) and strategic prosocial decision (i.e., decision to give with an opportunity to gain extrinsic reward). Both types of prosocial decisions commonly activated the reward system, while altruistic prosocial decision additionally recruited the sgACC and the posterior VMPFC. Given the role of these regions in affiliative behaviors, this result suggests that prosocial behavior with altruistic motivation may be intrinsically rewarding due to its function in the formation and maintenance of social bonding. Together, these findings indicate that improving the welfare of others can have different subjective values and rewarding qualities depending on individual differences, situational or cultural contexts, and the person-situation interaction.

## Conclusion

An overview of the social neuroscience literature focusing on social connectedness and wellbeing has yielded a number of converging results. First, both beneficial (e.g., social acceptance) and detrimental (e.g., social isolation) effects of social connectedness on wellbeing co-occurred with changes in the subcortical reward system, such that the former enhanced, whereas the latter suppressed its activity. Second, activity of the central nodes of the social brain, most notably the MPFC and ACC, is modulated by factors influencing social connectedness and wellbeing. It is worth noting that more recent fMRI work tended to examine these nodes as a part of a larger functional network rather than isolated regions, such as the default mode network. In order to shed further light on the possible mechanistic link between social connectedness and wellbeing that extends to more general health outcomes (2), future studies would benefit from harnessing meaningful individual differences in the social brain by studying different populations.

## Author contributions

MJK and SS reviewed the relevant literature and wrote the manuscript. All authors contributed to the article and approved the submitted version.
